# Artificial Neural Network-Based Mechanism to Detect Security Threats in Wireless Sensor Networks

**DOI:** 10.3390/s24051641

**Published:** 2024-03-02

**Authors:** Shafiullah Khan, Muhammad Altaf Khan, Noha Alnazzawi

**Affiliations:** 1 College of Computing and Systems, Abdullah Al Salem University, Kuwait City 72303, Kuwait; 2 Institute of Computing, Kohat University of Science and Technology, Kohat 26000, Pakistan; 3 Department of Computer Science and Engineering, Yanbu Industrial College, Royal Commission for Jubail and Yanbu, Yanbu Industrial City 41912, Saudi Arabia

**Keywords:** wireless sensor network, artificial neural networks, backpropagation, routing attacks

## Abstract

Wireless sensor networks (WSNs) are essential in many areas, from healthcare to environmental monitoring. However, WSNs are vulnerable to routing attacks that might jeopardize network performance and data integrity due to their inherent vulnerabilities. This work suggests a unique method for enhancing WSN security through the detection of routing threats using feed-forward artificial neural networks (ANNs). The proposed solution makes use of ANNs’ learning capabilities to model the network’s dynamic behavior and recognize routing attacks like black-hole, gray-hole, and wormhole attacks. CICIDS2017 is a heterogeneous dataset that was used to train and test the proposed system in order to guarantee its robustness and adaptability. The system’s ability to recognize both known and novel attack patterns enhances its efficacy in real-world deployment. Experimental assessments using an NS2 simulator show how well the proposed method works to improve routing protocol security. The proposed system’s performance was assessed using a confusion matrix. The simulation and analysis demonstrated how much better the proposed system performs compared to the existing methods for routing attack detection. With an average detection rate of 99.21% and a high accuracy of 99.49%, the proposed system minimizes the rate of false positives. The study advances secure communication in WSNs and provides a reliable means of protecting sensitive data in resource-constrained settings.

## 1. Introduction

Human developments invariably have certain shortcomings [1]. All the progress made in the realm of communication, encompassing WSN, internet of things (IoT), embedded systems, ad hoc networking, and similar innovations, can be attributed to human ingenuity. In this context, human ingenuity is oriented towards enhancing lifestyle comfort through applications including smart homes, efficient transportation, and remote communication, among others. Currently, WSNs are widely embraced for sensing and controlling remote applications. A WSN is a network that consists of several sensor nodes strategically placed across a large region to monitor and record various physical parameters. Temperature, wind speed, pressure, humidity, and a variety of other characteristics are all scattered throughout multiple sites. Importantly, sensor nodes in a WSN are battery-powered and may operate in remote regions where human access is restricted or impossible [2].

In WSNs, numerous sensor nodes continuously capture data and transmit the data to a selected sink node. These data are transmitted either directly from node to node or through intermediaries known as cluster heads. The sensor nodes communicate their data to the cluster-head nodes, which then forward this information to the base station (BS) for further communication [3,4].

WSNs are structured with a layered design wherein each layer serves distinct functions critical for efficient data transmission and network operation. These layers, including the physical, data-link, network, transport, and application layers, play pivotal roles in ensuring the reliable and secure operation of WSNs [5].

At the physical layer, which constitutes the lowest layer of the WSN architecture, the network faces several security challenges. Eavesdropping attacks, compromised node attacks, replication node attacks, and basic jamming attacks are primary concerns in this layer. These threats target the fundamental components of the network, such as nodes and communication channels, potentially compromising data integrity and confidentiality. At the data-link layer, attacks such as denial of service (DoS) attacks, collision attacks, unfairness attacks, and intelligent jamming are prominent concerns. This layer is responsible for managing communication between neighboring nodes and addressing issues related to data packet collision and channel access. Ensuring the integrity of data-link-layer protocols is crucial to maintenance of network reliability.

The network layer introduces further security challenges, including sybil attacks, sinkhole attacks, spoofing attacks, and black-hole, gray-hole, and wormhole attacks. These attacks exploit vulnerabilities in routing protocols and can disrupt data transmission, hijack routing paths, or impersonate legitimate nodes, posing significant threats to network functionality. The transport layer is susceptible to attacks like flooding and desynchronization, which can impact the quality of service and the reliability of data delivery. This layer manages end-to-end communication and ensures data integrity, making it a crucial aspect of WSN security.

Lastly, at the application layer, data collected by WSNs are processed and utilized for various applications. Ensuring the security and privacy of this data is essential. Attackers may attempt to manipulate or intercept application-layer data, necessitating the use of robust security measures to safeguard sensitive information. 

WSNs have emerged as a vital technology for a wide range of applications, from environmental monitoring and healthcare to industrial automation and surveillance. These networks consist of numerous small, self-governing sensor nodes that collaborate to collect and transmit data wirelessly, facilitating efficient data collection and dissemination. However, the distinct attributes of WSNs, including their limited resources, dynamic network topology, and vulnerability to diverse attacks, expose them to risks, particularly at the network layer. 

**Figure 1:**
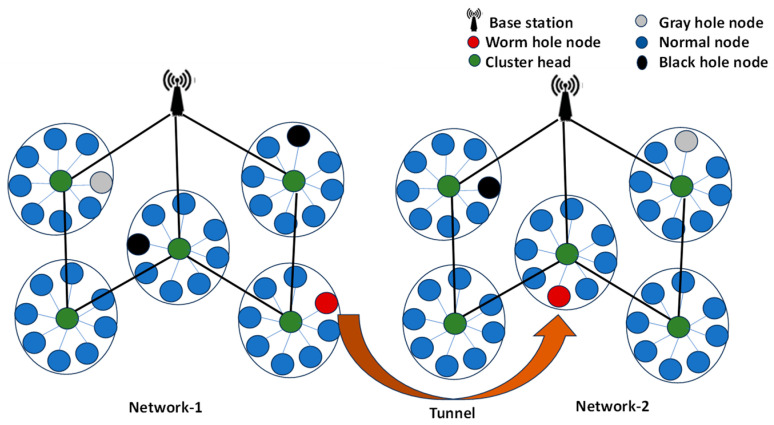
Routing attacks in a wireless sensor network.

The network layer within a WSN bears the responsibility of establishing and maintaining communication pathways between sensor nodes, enabling the routing of data from its source to the intended destination. Despite its essential role, the network layer is vulnerable to various security threats that imperil the integrity, confidentiality, and accessibility of data. In hop-to-hop communication in WSNs, a node always prefers the path towards the destination that includes fewer hops, which is called the optimal path. Due to the shorter distance, the optimal path provides many benefits like lower transmission overhead, lower energy consumption and less packet loss. When a node in multi-hop communication requests the path towards the destination, attacker nodes will present themselves as the nodes closest to the destination. The requesting node will select the path offered by the attacker node due to optimal path selection, and a routing attack then occurs in the WSN. Routing attacks manipulate the routing process to disrupt the accurate transmission of data, leading to network congestion, data loss, and possible denial of service. These attacks on the network layer come in several forms, such as flooding attacks, spoofing attacks, sybil attacks, black-hole attacks, gray-hole attacks, sink-hole attacks, and wormhole attacks [6]. Some of these types of attack are shown in Figure 1.

Flooding attacks occur when an excessive number of route requests is generated within a network. These attacks can be highly damaging, depleting network resources, reducing availability, and overburdening nodes, preventing them from fulfilling their tasks [7].

The primary objective of a spoofing attack is to create a cyclic path between source and destination nodes within WSNs by introducing false routing information. Such an attack possesses the ability to manipulate routing data. This type of attack can result in severe consequences, including node partition, the redirection of nodes along incorrect paths, a reduction in network lifetime, and the dissemination of erroneous routing information [8].

A sybil attack involves an attacker forwarding multiple messages with different IDs to various nodes while making them appear as if they are coming from different sources. The attack thus causes collisions in the network, forcing nodes to find alternate routes. The attacker manipulates this situation to create confusion among nodes, making it challenging for them to select the correct path for data transmission. This type of attack can disrupt data integrity and alter the data flow by changing the routing path [9].

A rogue node in the network fabricates a black-hole attack by drawing all traffic to itself while claiming to have the shortest path to the target. Meanwhile, the packets are absorbed or dropped by the malicious node, preventing them from reaching their intended destination. The intention is to cause a “black hole” in the network that will hinder data from reaching its intended destination and obstruct communication. 

Gray-hole attacks, also known as selective forwarding attacks, are more intricate, as compromised nodes claim to have superior routes in order to attract data packets, with the aim of subsequently dropping or modifying the packets before they reach their destination. This attack subverts the routing process, potentially allowing unauthorized access to sensitive information. 

In wormhole attack, two or more nasty nodes band together through either high-speed wireless communication or ethernet or fiber-optic cables to form a tunnel that connects them. By swiftly moving packets from one end of the network to the other, the tunnel creates the illusion that nodes are closer to one another than they are. The attackers’ goal is to establish a shortcut in the network, which could lead to nodes choosing the wrong route and possibly interfere with communication. Because of their transparent operation, wormhole nodes are invisible to other nodes in the network. As a result, they operate seamlessly and do not require cryptographic keys or network IDs [10].

To counter these threats, numerous preventive strategies have been proposed to fortify the security of the network layer within WSNs [10,11,12]. These strategies encompass a variety of techniques, including authentication, intrusion-detection systems, and secure routing protocols. Authentication mechanisms ensure that nodes interact solely with authenticated and trusted counterparts, thereby preventing unauthorized nodes from connecting to the network. Intrusion-detection systems monitor network activity, identifying anomalous behavior patterns to promptly detect attacks and initiate appropriate countermeasures.

Critical to prevention, secure routing protocols focus on establishing secure and efficient pathways for data transmission. Protocols like low-energy adaptive clustering hierarchy (LEACH) and ad hoc on-demand distance vector (AODV) incorporate security features to safeguard against a multitude of attacks. This safeguarding ensures data confidentiality, integrity, and authenticity throughout the routing process, enhancing the overall security of the network layer in WSNs. In contrast to these traditional strategies, ANN-based techniques provide the best solution for intrusion detection at the network layer in WSNs. 

In contrast to traditional methods, ANN-based solutions offer a superior approach to detecting intrusions within WSNs at the network layer. Among these solutions, options like the feed-forward ANN technique and the convolutional neural network (CNN) model stand out. After evaluating various ANN-based models, we will select the most effective one for the detection of routing attack in WSNs.

## 2. Literature Review

A multi-layer architecture for intrusion detection in WSNs proposed in [13] improved network security. In this architecture, the sensors are positioned within the initial layer, situated at the periphery of the network. The subsequent layer is positioned at the network’s core. The researchers employed the I Bayes method, known for its straightforward and efficient computational approach, as the core classifier. The initial layer’s role involves categorizing observed data as normal or potentially harmful without delving into specific attack patterns. The intermediate detection layer resides within the cloud and exclusively processes anomalous activities. This positioning reduces resource constraints, allowing for the implementation of more intricate techniques and in-depth analysis. Subsequently, the identification of harmful traffic is accomplished using the random forest (RF) method alongside a multi-class classifier. This classification model can ascertain the nature of the attempted breach and offer recommendations for selecting an appropriate defense strategy.

The results clearly indicate that their proposed multi-layer security approach led to improved true-positive rate (TPR), true-negative rate (TNR), false-positive rate (FPR), and false-negative rate (FNR) values. Additionally, a high level of accuracy was achieved. The obtained values were 100%, 90.4%, 99.5%, 97%, and 99.9% for normal, flooding, scheduling, gray-hole, and black-hole attacks, respectively. In comparison, previous research yielded values of 99.8%, 90.4%, 99.5%, 91.1%, and 73% for normal, flooding, scheduling, gray-hole, and black-hole attacks.

The work done in [14] proposed a novel approach that combines an on-demand link and an energy-aware dynamic multipath (O-LEADM) routing system for mobile ad hoc networks (MANETs). This approach aimed to effectively detect black-hole nodes through the baiting strategy while also distinguishing between packet loss caused by congestion and packet loss caused by malicious nodes. The suggested technique demonstrates a notable capability to differentiate between packet drops that occur due to link failures and those triggered by malicious nodes. In their study, the researchers compared their O-LEADM–black-hole identification approach with the existing AODV–black-hole routing system. They then analyzed various metrics to evaluate the performance of both strategies. Specifically, with respect to packet-delivery ratio, the proposed O-LEADM approach surpasses the current AODV–black hole method. 

For instance, with 84% of nodes moving at a velocity of 20 m/s, the AODV–black hole strategy experiences a reduction to 83% as mobility velocity increases. In contrast, the O-LEADM–black-hole strategy maintains a delivery ratio of 87% even at 20 m/s. Furthermore, O-LEADM–black hole adjusts its delivery ratio as mobility speed increases, accounting for connectivity faults and channel accessibility in order to mitigate congestion and enhance route reliability. Examining average delay, the study revealed that the AODV–black hole approach exhibits an average delay of 0.083 M.Sec at higher mobility speeds. In comparison, the O-LEADM–black hole approach achieves a lower average delay of 0.050 M.Sec. The energy-consumption analysis (ECA) showrf that in the presence of an adversary, the OLEADM–black hole approach consumes an average of 6.0 Joul of energy when operating at higher speeds. On the other hand, the AODV–black hole approach expends 11.2 J of energy under similar conditions. The overhead of the O-LEADM–black hole strategy ranges between 4.1 and 11.1, whereas the AODV–black hole approach exhibits an overhead ranging between 5.9 J and 16.6 J.

Notably, the O-LEADM–black-hole approach displays significant improvement in identifying other channels when a black-hole node is detected. This effectiveness is achieved with substantial movement and minimal expense. In contrast, the route-identification process in the AODV–black-hole strategy incurs higher costs in terms of detecting alternative routes to the destination. 

A machine-learning classification algorithm proposed in [15] for the detection of distributed denial of service attacks, including flooding, gray-hole, and black-hole attacks, within wireless WSNs. The investigation was carried out utilizing a dataset specific to WSNs, denoted as WSN-DS. In this evaluation, both accuracy and speed were taken into consideration as performance metrics. The outcomes of the analysis revealed that the J48 algorithm proved to be the most accurate and expeditious means of identifying gray-hole and black-hole attacks. Simultaneously, the random tree method emerged as the most accurate and swift approach for detecting flooding attacks. Specifically, in terms of speed, the J48 algorithm demonstrates efficiency, demanding an average processing time of 0.54 s per sample.

In [16], an approach was proposed that consisted of four distinct components: network data collection (NDC), gray-hole detection (GD), gray-hole prevention (GP), and gray-hole reduction (GR). The GD and GR procedures are conducted separately during the execution of the proposed system. In contrast, execution of the remaining functionalities is dependent on the analysis of dependencies. Furthermore, the initiation of network data collection occurs exclusively when a network controller retrieves packets from nearby nodes. On the other hand, the prevention process is activated when a flow exceeds a specified threshold.

The work done in [17] introduced an innovative lightweight encryption-based technique designed to counter sinkhole attacks in mobile wireless sensor networks (MWSNs). The primary focus of this proposed approach was to concurrently minimize energy consumption and enhance the precision of sinkhole-attack prevention. To accomplish this, a hybrid encryption algorithm was employed that integrated the robust blowfish algorithm for message encryption. This inclusion ensured both data integrity and the security of MWSNs against sinkhole attacks. Notably, the blowfish algorithm was chosen due to its resource-efficient nature and rapid processing, which render it highly suitable for MWSNs. To ensure the secure exchange of the blowfish key between the sender and the receiver, a strategic protocol was devised. At the initiation of the process, the sender employs the RSA algorithm to encrypt the blowfish key. This cryptographic approach guarantees the confidentiality and reliability of the key-sharing process. Subsequently, the recipient can effortlessly decrypt the message by employing its proprietary private key, ensuring the secure exchange of sensitive information.

A hybrid spectrum management optimization (HSMO) technique was proposed in [18] for identifying and resolving jammed nodes within a network. This method involves the utilization of edge nodes, sub-edge nodes, and cluster heads to detect and address jammed nodes. These specific node types play a key role in identifying instances of node jamming.

The process begins with the edge nodes, sub-edge nodes, and cluster heads being selected to participate in the jammed-node-detection procedure. As a result of this step, these nodes collectively contribute to the identification of jammed nodes. The edge node’s role involves evaluating objective function 1 across packets received from the sub-edge node. This assessment occurs on distinct channels but with identical frequencies. The sub-edge node evaluates the second objective function based on signals received from the cluster head. In particular, it focuses on two consecutive packets it receives. If neither of these packets satisfies the stipulated objective function, the sub-edge node severs its connection with the cluster head, resulting in a loss of connectivity. The cluster head engages in the hybrid spectrum management optimization (HSMO) procedure to both detect and eliminate the identified jammed node. Through this method, the network aims to effectively manage instances of jamming and enhance the overall performance and reliability of the communication environment.

The work presented in [19] proposed that a cluster-head node should select some surveillance nodes to calculate reputation and trust routes. A monitoring mechanism and anomaly-detection mechanism based on energy thresholds are used to detect errors generated by the attacked node or invalid data. This study ignored the trust rating of CH, which is an obvious disadvantage of this plan. 

The authors [20] proposed a trust-based low-power routing protocol (TEESR) that uses an authorization mechanism to reduce the probability of multiple malicious nodes occurring in adjacent areas. The node trust value determines the coverage radius and multi-path security route, and then the cluster head and the base station node make a comprehensive judgment to select the route with the higher security value. Although the proposed protocol performs well in dealing with external attacks, such as convergence holes and wormholes, it is powerless to deal with internal attacks.

A new trust-aware secure routing protocol (TSRP) was suggested in [21] to prevent wormhole, selective-forwarding, and black-hole attacks. The protocol has good performance in terms of average packet loss rate and throughput, but its high energy consumption is a disadvantage. 

The authors of [22] investigated the impact of black-hole denial-of-service attacks on the general-purpose ad hoc on-demand distance vector (AODV) protocol. The evaluation encompassed normal AODV, BH_AODV, and D_BH_AODV variations. These attacks were found to considerably impede network operations. Countermeasures such as intrusion-detection systems (IDS) and digital signatures were employed to thwart black-hole attacks. Quality of service (QoS) metrics, including packet delivery ratio (PDR), delay, and overhead, were adopted to conduct a comparative analysis among the conventional AODV, BH_AODV, and D_BH_AODV protocols. The assessment took into account varying factors such as nodes, packet sizes, and simulation durations. The experimentation employed NS2 to emulate malicious protocols and featured a customized D_BH_AODV routing protocol. The results revealed that the D_BH_AODV approach substantially enhances PDR by 40% to 50% across different nodes and packets. Moreover, as the number of nodes and the number of packets increased, the delay was notably reduced, from 300 to 100 ms and from 150 to 50 ms, respectively. Node and packet configurations influenced overhead levels, which ranged from 1 to 3. The findings underscore the detrimental impact of black-hole attacks on network performance. However, the introduced D_BH_AODV strategy demonstrated efficacy in enhancing QoS. This improvement was achieved through the identification and circumvention of black-hole nodes, which effectively ensures more secure and reliable communication pathways.

An effective approach proposed in [6] was used to identify black-hole attacks with notable efficiency. The occurrence of a black-hole attack leads to notable enhancements in crucial network parameters such as TH, TND, NRL, PDR, and PLR. To achieve this enhancement, the method presented employs a combination of techniques, including the K-nearest neighbors (KNN) algorithm for clustering, the beta distribution, Josang’s mental logic, and fuzzy inference to compute trust levels. These calculated trust levels contribute to the establishment of node reputation, which is subsequently evaluated by a trust server. Ultimately, guided by the reputation table, the cluster master undertakes periodic actions.

A detailed analysis presented in [23] focused on performing a forensic examination of ad hoc IoT networks that employ the AODV routing protocol. This analysis was conducted within the context of black-hole attacks, a form of denial-of-service attack that poses a substantial threat to IoT networks. The investigation also evaluated patterns in network traffic and node behavior, aiming to quantify the impact of these attacks. To support digital forensic (DF) inquiries, the vulnerabilities inherent in the protocol were systematically scrutinized. The research involved the reconstruction of networks across various operational modes and parameters. This reconstruction served to validate the analysis conducted and to propose recommendations for the development of robust routing protocols. The overarching goal was to enhance understanding of IoT network performance in the face of black-hole attacks and to thereby contribute to the enhancement of more secure and resilient routing protocols.

A study discussed in [24] introduced a robust path-selection mechanism tailored to enhance the security and reliability of WSNs. The core purpose was to proactively identify and counter black-hole attacks, thus averting the compromise of routing nodes. By optimizing the routing of data packets through more efficient paths, the primary aim was to extend the operational lifespan of sensor nodes within WSNs. To fulfill this objective, the research proposed a trust-based route-selection mechanism that ensures the integrity of data transmission by identifying dependable routes between source and destination nodes. The process involves generating a source node and facilitating secure data transmission to the intended recipient. A trust-based assessment detects malicious nodes by evaluating their trust values. Once a malicious node is detected and surpasses a predefined threshold, a different, secure routing path is established for data transmission.

Throughout the data-transmission process, the trust-based path-selection technique is used, guiding the selection of secure routing paths. This strategy effectively safeguards the network against threats like black-hole attacks and selective forwarding, bolstering overall security and data integrity. The mechanism effectively integrates the trust system across various dimensions, ensuring the security of routing paths and the identification of optimal threshold values grounded in trust considerations. These aspects have a twofold impact: they influence the trust system’s performance and shape the threshold-based trust parameters. To realize these objectives, the mechanism computes a distinct threshold value for each potential path. This computation aims to pinpoint the path with the lowest threshold value, denoting the most secure route for data transmission. Consequently, at each step of detection and data-packet transmission, sensor nodes can gauge their level of trust, mitigating potential threats like black-hole attacks and selective forwarding.

In [25], an approach to enhance network security against potential attacks from internet traffic intruders was presented. The proposed solution aimed to strike a balance between the imperative for protection and the constraints posed by available resources and trust factors. To achieve this, the research introduced a ranking-based route mutation mechanism that leverages the bafflement technique for selecting optimal routes for flows within the network. These routes are chosen based on diverse considerations, ensuring robust security at the base station level by incorporating factors such as route overlap, energy consumption, and link cost. Moreover, this strategy supports multiple altered pathways that can confuse potential attackers, along with scrutiny algorithms tailored for fully centered WSNs. The technique involves strategically selecting multiple intruder sink nodes, which are designed to mislead attackers attempting to locate the true sink node. A suitable parameter is employed to account for the residual energy of neighboring nodes associated with the chosen intruder sink nodes. This parameter takes into consideration the expected additional communication cost within the corresponding region. Notably, this solution remains cost-effective and suitable for deployment in extensive sensor networks without imposing excessive expenses.

Another mechanism presented in [26] involves the implementation of a black-hole attack and introduces a corresponding prevention method for the AODV routing protocol within WSNs. The proposed prevention technique is centered on identifying the shortest paths between a non-compromised source and destination, as depicted in the diagram. It is important to note that both the source and destination nodes are presumed to be trustworthy and not compromised. Our approach involves incorporating the detection of malicious paths within the route-discovery phase of the AODV routing protocol.

A fuzzy-logic-based technique was proposed in [27] to tackle the issue of gray-hole attacks targeting WSNs nodes integrated into IoT systems. The IoT system incorporates WSN by establishing connectivity through a router to amass data. By leveraging the deployed sensors, the nodes can detect attacks within the IoT framework. Each node within the IoT network is systematically organized, serving various functions. This organization is underpinned by connecting users and clients to a central controller, the base station, facilitating IoT connectivity. The intricate architecture intrinsic to IoT renders it susceptible to gray-hole attacks. The sensor nodes situated within the WSN play a pivotal role in detecting attacks occurring within the IoT domain.

Routing protocols are pivotal in both WSN and IoT, effectively guiding the decision-making process for packet forwarding among nodes. Within the realms of IoT and WSN, two primary categories of routing protocols exist: proactive and reactive. Proactive routing protocols process network information, while reactive routing protocols manipulate the network’s structure to facilitate efficient packet routing.

In [28], a system for detecting intruders in WSNs and responding with an intelligent mobile robot was described. This system employs an unsupervised neural network for intrusion detection, focusing on identifying changes over time using a Markov model. Once an intrusion is detected, a robot is dispatched to the affected area for further investigation. The reported results indicate an approximate detection rate of 85%.

Given that WSN nodes are often deployed in harsh and unattended environments, where attackers may compromise a subset of nodes, compromised sensor nodes can potentially transmit incorrect data to the central sink. To address this concern, a malicious-node-detection mechanism is proposed in [29] for hierarchical WSNs, where nodes gather and share data with neighboring nodes. This mechanism utilizes a feed-forward ANN technique. The authors assert that their proposed approach effectively identifies malicious nodes, even when up to 25% of sensor nodes have been compromised.

In [30], the author placed a significant emphasis on addressing the challenge of detecting data anomalies in WSN. To tackle this issue, a convolutional neural network (CNN) model was meticulously crafted, leveraging the distinctive features of the marked mode and the deep neural network architecture to effectively identify anomalous data patterns. Throughout the study, the author introduced three innovative network models and conducts a comparative evaluation against a previously utilized CART (classification and regression trees) model. The assessment of these models was based on performance metrics such as detection accuracy (DA), true-positive rate (TPR), and precision (PRE). The experimental findings unequivocally demonstrate that the three models introduced in this research consistently outperform the CART model, with the M2 model exhibiting the highest level of performance.

The author of [31] introduced a novel approach by utilizing a hybrid ANN method to identify uncommon elements within the framework of a “smart home” system. This research marked the first instance of employing a hybrid ANN technique in the domain of detecting irregularities within “smart home” or building-automation systems. The outcomes of the experiment demonstrated that the ANN outperforms conventional machine learning methods, achieving an impressive area under the ROC curve of 0.9689.

A hybrid approach proposed in [32] combined the definition of fuzzy logic with the learning ability of neural networks to detect routing attacks in WSNs. The success of this solution depends on the quality and quantity of training data, the creation of fuzzy rules, the neural network architecture and the starting point. To ensure that the system effectively detects and mitigates attacks in a dynamic network environment, regular maintenance and development are required.

A comprehensive overview of the proposed mechanisms discussed in this section is given in Table 1.

## 3. Artificial Neural Networks 

**Figure 2:**
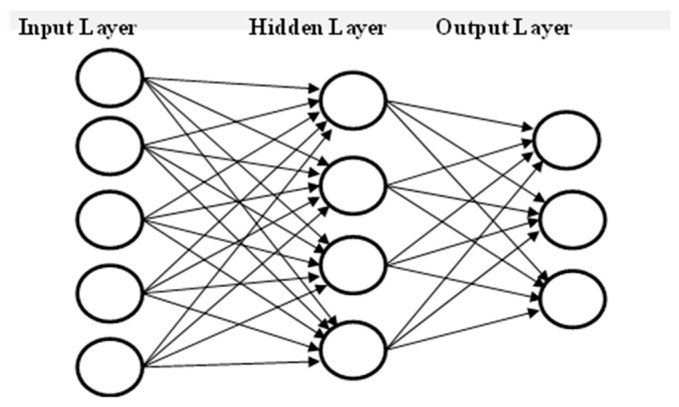
General artificial neural network architecture.

ANNs are based on the concept of the biological neural network. An ANN is composed of a large number of highly interconnected neurons arranged into different layers, i.e., the input layer (IL), the hidden layer (HL) and the output layer (OL), to provide the desired solution to a problem, as shown in Figure 2. These layers are connected through weighted connections. The network gains the ability to modify the weights attached to each connection between nodes to enhance its performance on a particular job. An ANN’s performance may be impacted by the quantity of the HL and neurons within them. Although having many neurons in the HL or HLs may increase network complexity, it may also guarantee accurate learning. There are two types of ANN: recurrent neural networks (RNNs) and feed-forward ANNs (FNNs). In FNNs, information travels in only one direction, that is, from the IL through the HL to the OL. By contrast, in RNNs, the information travels in both directions by introducing loops into the network [33]. To train these networks, we employ two important supervised learning algorithms and/or unsupervised learning algorithms. In supervised learning, set of inputs and the expected outputs are supplied to the network during the training process. The error between the produced and required outputs is used to modify the weights of the connections. The quality of the training dataset determines how well the ANNs perform. ANNs using more accurate training datasets produce more accurate outcomes. 

For supervised-learning-based ANNs, data collection for training presents a significant challenge. When the intended outputs are unknown during training, unsupervised learning can be used because it does not require input for connection-weight adjustments. As there are no established categories for the classification of the output patterns, the system must create its own representations for the inputs that are provided. The most-often-used learning algorithm for classification in feed-forward ANNs is backpropagation (BP). In a BP algorithm, to reduce the discrepancy between the desired output and the projected output obtained during the forward pass, a gradient-based optimization approach is used to reduce the error in the outputs. This approach involves modifying the weights of the connections between the neurons in the hidden and output layers during the backward pass.

## 4. Proposed Methodology 

Our proposed ANN-based mechanism is composed of three layers. Because obtaining real datasets for WSNs is difficult, we used the CICIDS2017 dataset [34], provided by Canadian Institute of Cybersecurity, to train and test the proposed model. This dataset contains most of the currently relevant attack scenarios, which were enough to evaluate the effectiveness of our proposed system. The proposed model was trained with the four features extracted from CICIDS2017. These parameters are as follows: number of packets received and forwarded by the node, energy-consumption details of the node [14], and the trust value of the node [19]. We had four nodes in the input layer. During training, various numbers of nodes in the hidden layer and various number of hidden layers were evaluated; however, the false-detection rate was determined to be low when there was one hidden layer that contained four nodes. As we are interested in the detection of three specific routing attacks due to their high-severity impacts, four nodes were used to make up the output layer. This layer detects whether the node is normal or malicious; a malicious node is involved in launching black-hole, gray-hole, wormhole attacks using the following outputs: [1, 0, 0, 0], [0, 1, 0, 0], [0, 0, 1, 0], and [0, 0, 0, 1], respectively. The NS2 simulator was used to obtain the results, which consist of the detection of the intended routing attacks.

Our ANN-based proposed technique operates in two parts. The first part involves training the system using the dataset that was obtained; the second part involves testing the trained system using actual traffic that was obtained via a network interface. When effectively trained with both normal and attack traffic, ANN-based algorithms yield optimal outcomes [35]. The dataset employed in our suggested mechanism has 20,000 input vectors, of which 15,000 are used for training, 3000 input vectors are used for testing, and the remaining 2000 input vectors are used to validate the proposed detection system. 

### 4.1. Training

**Figure 3:**
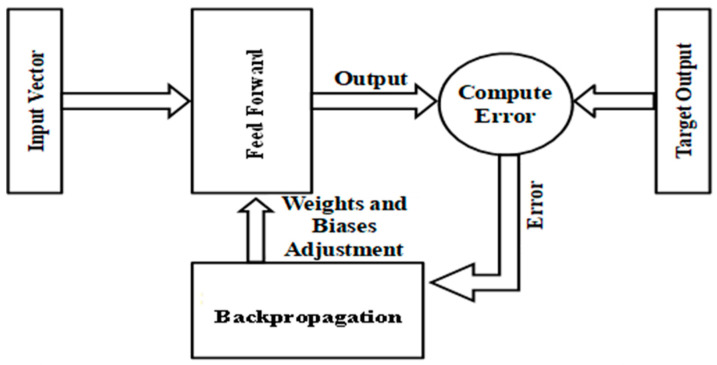
Training of the proposed mechanism.

The aim of training is to enable the system to learn how to classify nodes into the target category. The two stages of training for the proposed system are called feed-forward and back-propagation, as shown in Figure 3. 

#### 4.1.1. Feed-Forward

A vector of input values and the intended output value are sent to the system in a feed-forward configuration. The input vector of our proposed technique has the format {5, 5, 4, 1, 1, 0, 0, 0}, where {5, 5, 4, 1} indicates the number of packets the node has received and sent, the node’s energy consumption information, and the node’s trust value respectively mentioned in Table 2. The leftover values of the input vector, i.e., {1, 0, 0, 0} correspond to the desired output, which is “normal node” for the provided vector. The process of choosing initial weights and bias values is a significant problem, as these values aid in producing the intended results. This input vector is then forwarded to the hidden layer through connected weights. All implementation of the back-propagation algorithm can be found in [36].

(1) $${IH}_{k}=\sum_{l=1}^{4}\sum_{m=n}^{p}X_{l}W_{m}+b_{k}$$

In the above Equation (1), “*IH_k_*” stands for the scalar product of each input value times its linked weight and the total bias associated with each hidden layer neuron. The initial values of each variable in Equation (8) are as follows: *k* = [1,2,3]: *n* = 1, 5, 9, 13: *p* = 4, 8, 12, 16. The output of every node in the hidden layer is determined using the following sigmoid activation function:

(2) OHk=11+e−IHk 

In Equation (2), the output of each neuron in the hidden layer is denoted by “*OH_k_*”, and “*IH_k_*” is calculated in Equation (1). The result of Equation (2) for each hidden layer node will be used as input to each output layer node. The same process will be repeated to obtain the results from output layer nodes using Equations (3) and (4), as follows:

(3) $${IO}_{k}=\sum_{l=1}^{3}\sum_{m=n}^{p}{OH}_{l}W_{m}+b_{k}$$

(4) Outk=11+e−IOk

where “*Out_k_*” is the output of each output layer node for the input supplied by the hidden layer nodes “*OH*” and “*IO_k_*” is the sum of the bias associated with each output layer neuron and the scalar product of each input value received from the hidden layer times its linked weight. The variables in Equations (3) and (4) could have the values *k* = [1,2,3,4], *n* = 1, 4, 7, 10, and *p* = 3, 6, 9, 12.

The final stage in the suggested mechanism’s feed-forward training is to use the following equation to determine the error between the calculated output (*AO*) and the desired output (*DO*):

(5) $$E=\frac{1}{2}\sum_{i=1}^{4}{\left({DO}_{i}-{AO}_{i}\right)}^{2}$$

**Figure 4:**
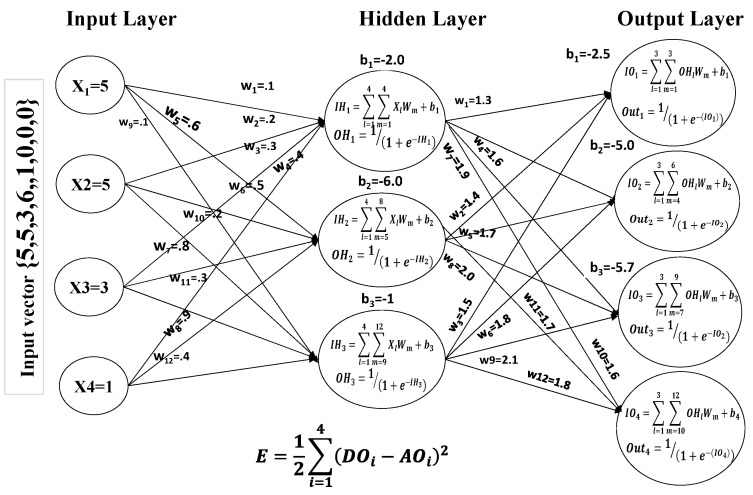
First forward pass of the first input vector.

During the various experiments performed for the identification of optimal error values, it was found that the system produced accurate results when the weights of connection and bias values were updated with the error value of 0.20. The first feed-forward mechanism for input vector {5, 5, 3, 1} is described in Figure 4.

#### 4.1.2. Backpropagation

If the error determined in the feed-forward stage is greater than 0.20, then the calculated error will be backpropagated to update the connected weights of each neuron in the hidden and input layers and the bias value associated with hidden-layer neurons until it satisfies the given error rate. Equation (6) is used in back-propagation to apply the difference in error generated by feed-forward to determine the gradient values for each node in the output layer.

(6) $${OGrad}_{i}={AO}_{i}\left(1-{AO}_{i}\right)*Error$$

Finding the rate of change in the hidden-to-output-layer-connection weights and the bias value associated with each output layer node is the next step in the back-propagation process. These rates of change can be calculated as follows:

(7) $$∆w_{ij}=LR*{OGrad}_{j}*{Hlo}_{i}$$

(8) $$∆b_{j}=LR*{OGrad}_{j}$$

The learning rate (*LR*), which controls how quickly the back-propagation algorithm learns, is used in Equations (7) and (8). Greater values of ‘*LR’* will cause ∆*w* to fluctuate more, increasing the possibility of overshooting a correct response. Thus, “0.02” is the value assigned to “*LR*” in our suggested process. Trial and error are used to determine this value of “*LR*”. Next, utilizing the obtained “∆*w_ij_*” and “∆*b_j_*”, the weights and biases related to the hidden to output layer are improved. After that, we used Equations (9)–(11) to calculate the gradient value, the rate of change in the input-weights-to-hidden-layer connections, and bias values for each node in the hidden layer, as follows:

(9) $${HGrad}_{i}={Hlo}_{i}\left(1-{Hlo}_{i}\right)*(\sum_{j=1}^{3}{OutGrad}_{j}*W_{j})$$

(10) $$∆w_{ki}=LR*{HGrad}_{i}*X_{k}$$

(11) $$∆b_{i}=LR*{HGrad}_{i}$$

The feed-forward will now begin anew. For the given vector, {5, 5, 3, 6}, feed-forward and back-propagation procedures will be performed until the generated gradient descent error reaches the desired error. 

**Figure 5:**
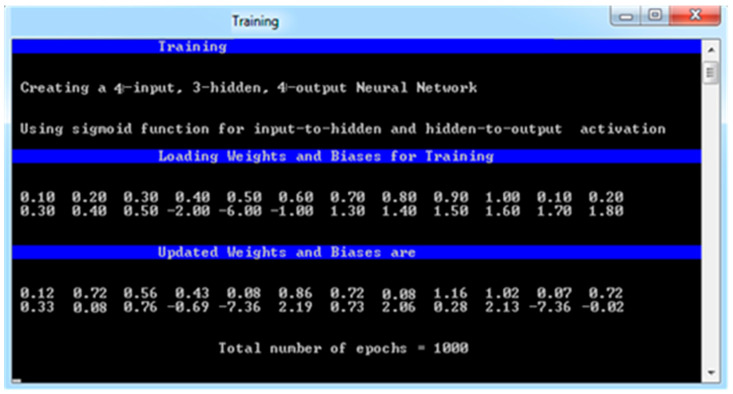
Generation of updated weights for input vectors.

After the updated weights and biases are generated, the training of the proposed system will be completed, shown in Figure 5. Following that, testing will be conducted using the remaining datasets of 3000 input vectors.

### 4.2. Testing

**Figure 6:**
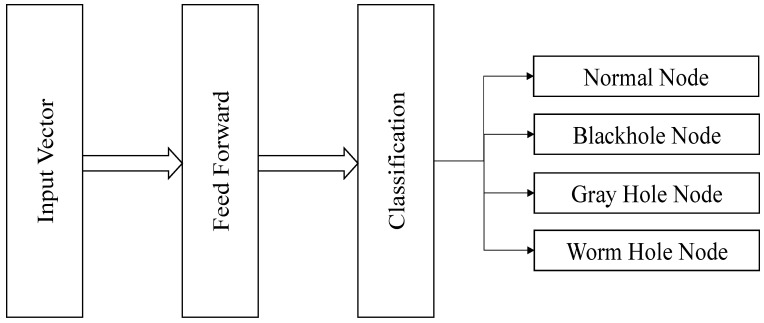
Testing of proposed mechanism.

After the system has been trained, the new updated weights and bias values are used by the system to classify the remaining 3000 input vectors of the dataset into their required category. During system testing, the values of learning rate, momentum, error rate and number of epochs are the same. Moreover, the same feed-forward steps used in training the system are followed in testing to generate the outputs from the output-layer nodes Figure 6. Unlike in training, in the testing phase, desired outputs are not provided with input vectors to the system. 

Following system training, the system uses the newly updated weights and bias values to categorize the dataset’s remaining 3000 input vectors into the appropriate categories. The same parameters for learning rate, momentum, error rate, and number of epochs are used for testing the system. Additionally, the same feed-forward procedures that were used to train the system are also used during testing to produce outputs from the output layer nodes. Unlike in training, the system does not receive input vectors for desired outputs during the testing phase.

## 5. Results and Discussion 

This section outlines and discusses the main finding of the proposed work. The number of packets sent and received (the packet delivery ratio), energy consumption and the trustworthiness of the node in the other network were the main criteria used for detection of malicious nodes in WSN in our proposed work. Network simulation (NS-2) was used to simulate and analyze the performance of the proposed detection system. Table 3 contains a list of the simulation parameters that were used in the experiments. The AODV routing protocol was employed in the experiment, and a 60-s simulation time is chosen. 

**Figure 7:**
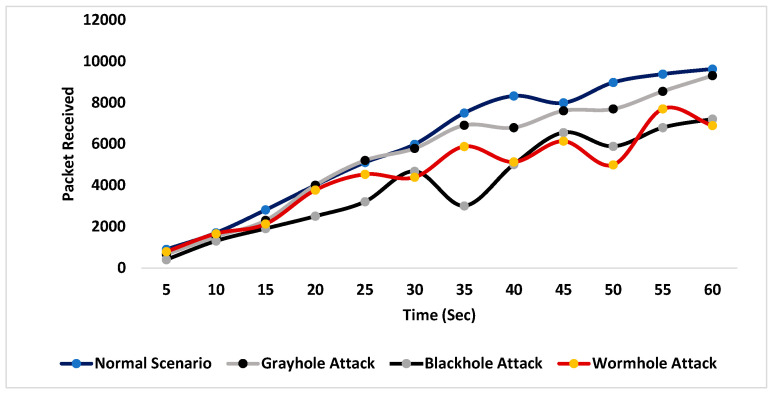
Packets received at the base station.

During the simulation, different scenarios were considered. Initially, no malicious node was used, and the number of packets received at the base station, the packet drop rate and the energy consumed by the network were recorded for 60 s. In the rest of the scenarios, the same parameters were noted during a black-hole attack, a gray-hole attack and a wormhole attack. The combined simulation results of packets received during each scenario are shown in Figure 7.

**Figure 8:**
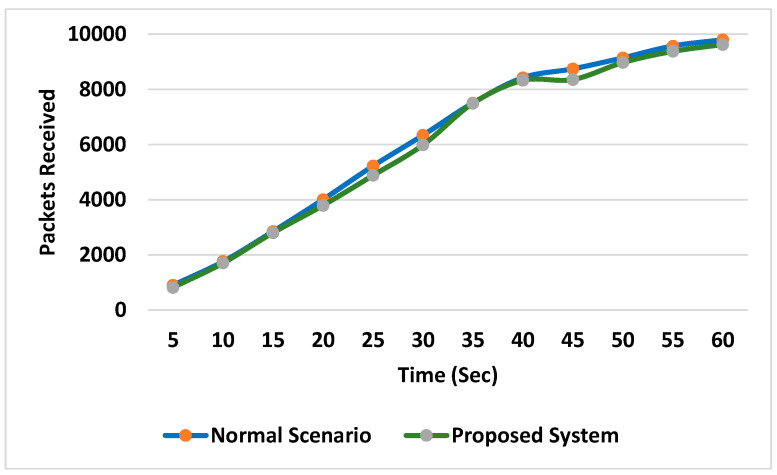
Comparison of the numbers of packets received at the base station.

In the last scenario, the proposed system was implemented, and all types of malicious nodes used in the previous scenarios, along with normal nodes, were used. Almost the same number of packets were received at the base station during all attacks as were received during the normal scenario (without attacks), as shown in Figure 8.

**Figure 9:**
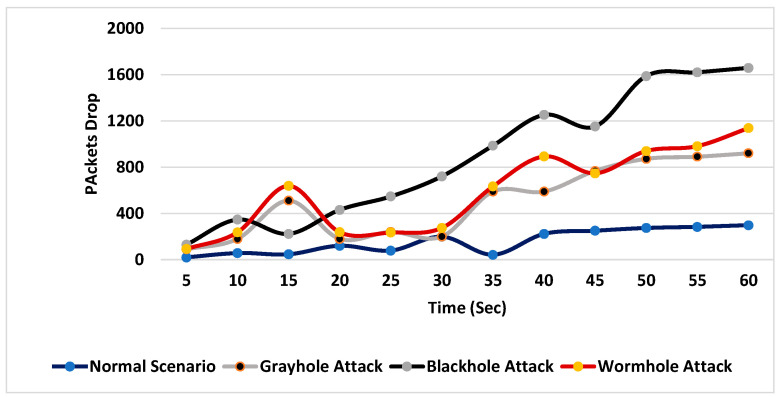
Packet drop rates without the proposed system.

**Figure 10:**
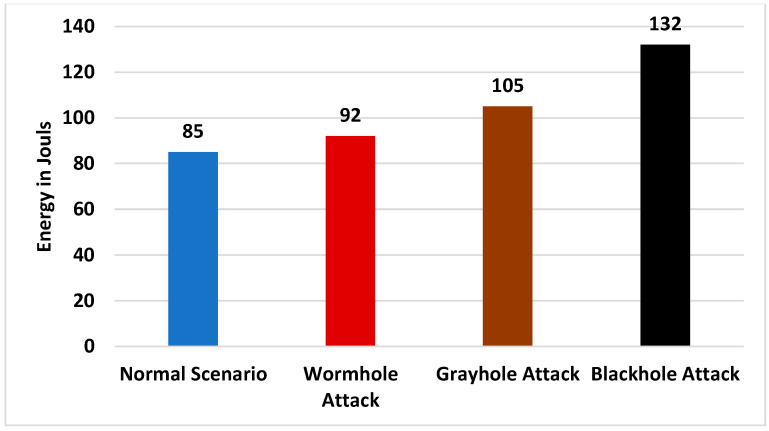
Energy consumption of the network without the proposed system.

Figure 9 and Figure 10 show that among all the scenarios, during the black-hole attack, the packet drop rate and the energy consumption of the network were especially high. 

**Figure 11:**
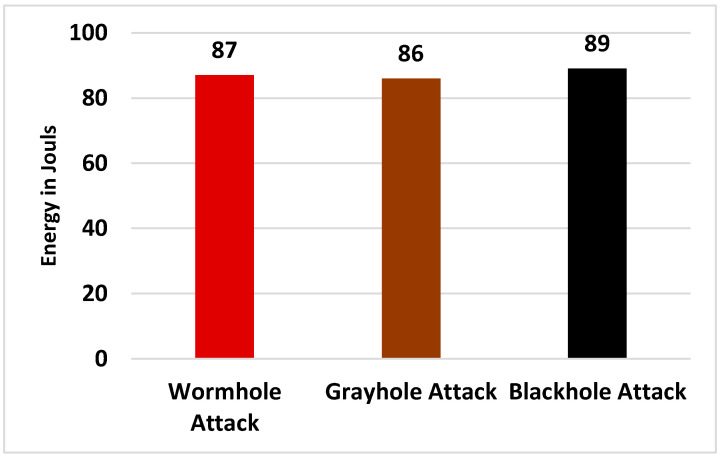
Energy consumption during routing attacks in the presence of the proposed system.

Figure 10 shows that energy consumption of the network was 85 J when there was no attack but that it gradually increased during routing attacks. The network consumed a very large amount of energy during the black-hole attack. When the proposed system was deployed and all the routing attacks were launched, the energy consumption of the network was almost like that of the network in the absence of the attacks, as shown in Figure 11.

**Figure 12:**
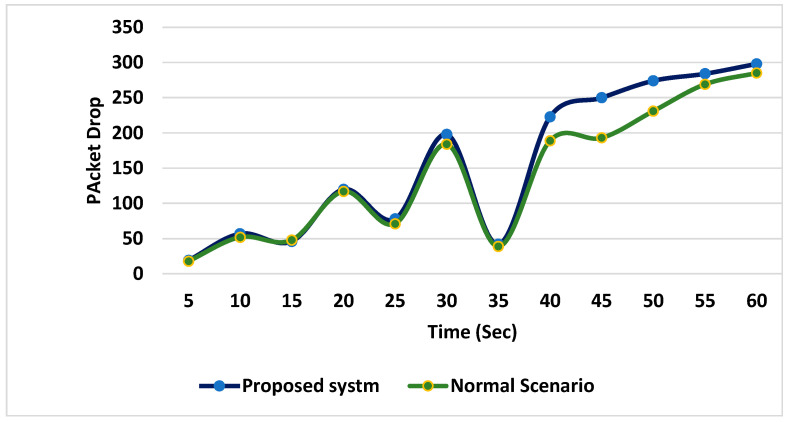
Packet drops rate comparison.

Moreover, when the proposed system was deployed and all the routing attacks were launched during the simulation, the rate of packet drop was very low due to early detection of malicious nodes, as shown in the Figure 12.

The proposed model’s performance indicators are contrasted with those of other machine learning algorithms like random forest (RF), decision trees (DT), and support vector machines (SVM). The analysis takes into account the following parameters: recall, accuracy, precision, F1-Score, and specificity. There are total 2000 input vectors in the dataset, which is composed of normal and abnormal data, with the abnormal data including three routing attacks. The complete dataset was split in a ratio of 75:15:10 in order to verify the proposed model’s detection rate and accuracy rate. Whereas for training we used 75% input vectors, in testing, we used 15% data, with 10% input vectors from the dataset used for validation. Table 4 lists the distribution of datasets for all attacks and normal scenarios.

The performance of the proposed system during the testing phase was evaluated using a confusion matrix, shown in Table 5. Results are classified as true positive (*TP*), true negative (*TN*), false positive (*FP*), and false negative (*FN*) in our confusion matrix. The examples are labeled “Normal” for a normal scenario, “B.H.” for a black-hole attack, “G.H.” for a gray-hole attack, and “W.H.” for a wormhole attack in order to make the analysis simpler. 

Performance measures such as detection rate (*DR*), false-positive rate (*FPR*), accuracy, and precision given in Table 6 are derived from the confusion-matrix values and examined for various attack scenarios. These matrices have the following mathematical formulation [32]:

(12) $$DR\;or\;TPR=\frac{TP}{TP+FN}$$

The number of correctly identified attacks divided by the total number of attacks yields the detection rate (*DR*). It is extremely rare for a classifier to obtain a *TPR* of 1, which indicates that every intrusion is successfully recognized.

(13) $$FPR=\frac{FP}{FP+TN}$$

The number of normal instances that are classified as attacks (*FP*) is divided by the total number of normal instances to obtain the *FPR*.

(14) $$Accuracy=\frac{TP+TN}{TP+TN+FP+FN}$$

Accuracy, also known as classification rate (CR), measures how well the classifier can distinguish between normal and anomalous traffic behavior. It is calculated as a percentage by dividing the total number of occurrences properly classified using baseline behavior features by all instances.

(15) $$Precision=\frac{TP}{TP+FP}$$

Precision measures how well a classifier makes positive predictions. For a given class, it is computed as the ratio of true positives to the total of true positives and false positives.

(16) $$F1Score=2*\frac{\left(DR*Precision\right)}{\left(DR+Precision\right)}$$

The *F*1 *Score* provides a balance between precision and *DR* by taking the harmonic mean of these two criteria. This score is used when it is desirable to take into account false positives and false negatives equally.

**Figure 13:**
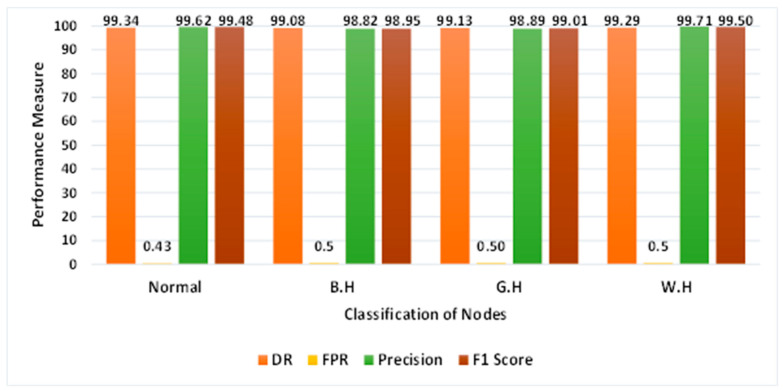
Performance evaluation of the proposed system.

Table 7 and Figure 13 show the performance analysis of the proposed system. It has been noted that practically every kind of attack is appropriately classified.

**Figure 14:**
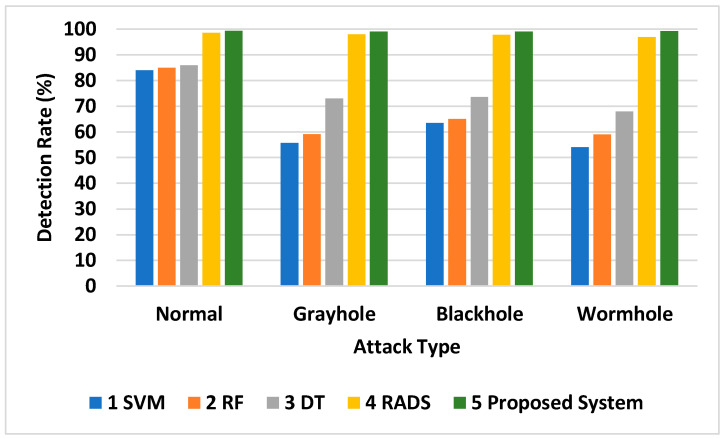
Detection rate Comparison.

**Figure 15:**
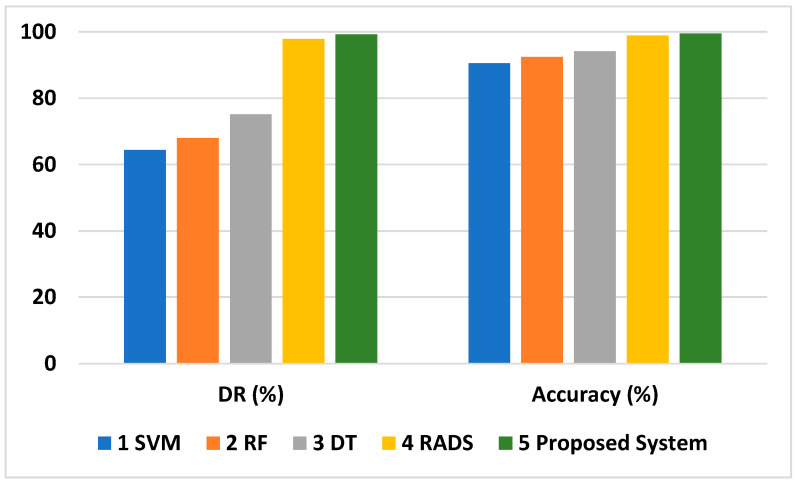
Comparative analysis of average detection rate and accuracy.

Feature selection results in the fewest incorrect classifications, yet the proposed system performs better than other detection methods, as shown by the analysis in Figure 14. This analysis compares the detection rate of different routing attacks by the proposed system with that of a technique proposed in [32] and those of machine-learning techniques like support-vector machines (SVM), DT, and RF. It is observed from the results that the maximum detection performance is attained by the proposed approach. The average detection rate of the proposed model is 99.21%, which is 1.38% greater than that of the approach proposed in [32], i.e., 97.8%. With regard to other machine-learning approaches, the average detection rate of our proposed system was 34.9%, 24.16%, and 31.26% better than the detection rates of SVM, DT, and RF, respectively, as shown in Table 8 and Figure 15.

The accuracy rate of the proposed system is also better than those of the other systems described in Figure 15 and Table 8. The improved performance is achieved due to the assortment of efficient features, the selection of initial values of the weights and bias values, and the number of neurons in the hidden layer used to train the proposed model.

## 6. Conclusions

In this work, we proposed a feed-forward neural network for detection of the most important routing attacks in the field of WSNs, with training and testing conducted using the CICIDS2017 dataset. A confusion matrix was used for analysis of the proposed system. The system’s detection rate of 99.21%, with a 99.45% accuracy rate, as shown by the confusion matrix, demonstrates its capacity to correctly detect and categorize the great majority of routing attacks. The system’s dependability in reducing misclassifications and guaranteeing that legitimate network activity is not tagged as harmful is further highlighted by the low false-positive rate. Simulation and analysis results proved that proposed system detected these attacks efficiently; as a result, quality of service and overall network performance improved.

The results of this study not only confirm the efficacy of our approach, but also make a valuable contribution to the development of secure communication in WSNs. In order to further improve the system’s performance, we plan in the future to investigate additional features and routing attacks by using advanced ANNs to extend our approach to real-world deployments, aiming for increased performance and reduced computation time.

## Figures and Tables

**Table 1:** Overview of literature.

Year	Author Name	Attack Type	Prevention	Benefits
2021 [13]	Alruhaily and Ibrahim	Multi-layer intrusion detection in WSNs	Naive Bayes classifier, cloud-based detection, random forest classifier	Improved network security, enhanced true-positive rate (TPR), true-negative rate (TNR), false-positive rate (FPR), and false-negative rate (FNR), increased accuracy.
2022 [14]	Suma and Harsoor	Black-hole detection using O-LEADM	On-demand link and energy-aware dynamic multipath routing system for MANETs	Enhanced black-hole node detection, differentiation between packet loss due to congestion and packet loss due to malicious nodes.
2022 [15]	Wazirali, R., & Ahmad, R.	DDoS detection	Machine learning-classification algorithms	Detection of flooding, gray-hole, and black-hole attacks, improved accuracy and speed.
2022 [16]	Gowdham Chinnaraju and S. Nithyanandam	Grey-hole detection and prevention	Network data collection, grey-hole detection, grey-hole prevention gray-hole reduction	Efficient gray-hole detection, prevention, and reduction mechanism.
			Prevention, gray-hole reduction	
2023 [17]	Alomirah, Y. A.	Sinkhole attack in MWSNs	Lightweight encryption-based technique, hybrid encryption algorithm, secure key exchange	Counteraction of sinkhole attacks, minimized energy consumption, enhanced security.
2023 [18]	Murugaveni, S., & Priyalakshmi, B.	Jammed-node detection	Hybrid spectrum management optimization (HSMO) technique	Effective identification and resolution of jammed nodes, improved performance and reliability.
2021 [19]	Ahmad F, Kurugollu F	Cluster-based DoS detection	Surveillance nodes, monitoring and anomaly detection mechanism, energy threshold	Utilizing cluster-head nodes for reputation and trust route calculation.
2020 [20]	Ilyas M, Ullah Z, Khan F A	Trust-based routing protocol	Trust-based low-power routing protocol (TEESR)	Enhanced security, authorization mechanism, focus on external attacks.
2021 [21]	Hu H, Han Y, Wang H	Trust-aware secure routing protocol	Trust-aware secure routing protocol (TSRP)	Prevention of wormhole, selective forwarding, and black-hole attacks. High performance in packet loss rate.
2021 [22]	Md Ibrahim Talukdar, Rosilah Hassan	Black-hole attack	Intrusion detection, digital signatures	Mitigation of black-hole attacks in AODV protocol, improved QoS metrics.
2021 [6]	Gholamreza Farahani	Black-hole attack detection and mitigation	K-nearest neighbors (KNN) algorithm, beta distribution, mental logic, fuzzy inference, trust server	Efficient black hole attack identification, enhanced network parameters.
2021 [23]	Hasan, A.; Khan, M.A.; Shabir, A.	Forensic examination using AODV Protocol	Examination of IoT networks with AODV protocol, vulnerability analysis, reconstruction of networks	Quantifying impact of black hole attacks, enhancing understanding of network performance.
2023 [24]	Mohammad Sirajuddin et al.	Secure-path selection	Trust-based route selection mechanism, optimized routing	Enhanced security, reliability, and operational lifespan of WSNs.
2023 [25]	E. Kavitha and S Sowndeswari	Network security against attacks	Ranking-based route mutation mechanism, bafflement technique, intruder sink nodes	Enhanced security against internet traffic intruders, robust security at the base-station level.
20223 [32]	M. Ezhilarasi, L. Gnanaprasanambikai, et al.	Routing-attack detection in WSNs	Fuzzy and feed-forward neural networks	Enhanced detection and accuracy rates

**Table 2:** Input vector parameters.

Attack Type	Input Vector Parameter for ANN
Black-hole attack, gray-hole attack and wormhole attack	Number of packets received by the node
	Number of packets sent by the node
	Energy-consumption details
	Trust value of the node in the network

**Table 3:** Simulation Parameters.

S.No	Parameters	Range/Value
1	Area	1000 × 1000 m
2	Nodes	500
3	BS location	1300–1400 m
4	Initial energy	1.5 J
5	Trust	1 or 0
6	Routing Protocol	AODV
7	Simulation Time	120 s
8	Bandwidth	25 Kbps
9	Transmission range	50 m
10	Packet size	512 Bytes

**Table 4:** Distribution of input vectors in the dataset.

Detail	Training	Validation	Testing	Total
Normal	4250	550	810	5610
Black-hole	3580	485	760	4825
Gray-hole	3550	475	720	4745
Wormhole	3620	490	710	4820
Total	**15,000**	**2000**	**3000**	**20,000**

**Table 5:** Confusion Matrix.

**Actual Class**	**Predicted Class**					
	**S.No**	**Classes**	**Normal**	**B.H**	**G.H**	**W.H**
	**1**	**Normal**	**806**	**1**	**2**	**1**
	**2**	**B.H**	**1**	**755**	**3**	**1**
	**3**	**G.H**	**1**	**2**	**716**	**1**
	**4**	**W.H**	**1**	**1**	**3**	**705**
	**Precision**		**99.63**	**99.47**	**98.90**	**99.58**

**Table 6:** Performance evaluation of the proposed system.

S.No	Class	*DR*	*FPR*	*Precision*	*F1Score*	*Accuracy*
1	Normal	99.34	0.43	99.62	99.48	99.49
2	B.H	99.08	0.50	98.82	98.95	
3	G.H	99.13	0.50	98.89	99.01	
4	W.H	99.29	0.50	99.71	99.50	
5	Average	99.21	0.48	99.26	99.23	

**Table 7:** Comparison of detection rates of routing attacks.

S.No	Techniques	Normal	Gray Hole	Black Hole
1	SVM	84	55.7	63.6
2	RF	85	59.1	65
3	DT	86	73	73.6
4	[32]	98.5	98	97.8
5	**Proposed system**	**99.34**	**99.13**	**99.08**

**Table 8:** Performance comparison.

S.No	Technique	*DR* (%)	*Accuracy* (%)
1.	SVM	64.33	90.51
2.	RF	68.02	92.42
3.	DT	75.16	94.11
4.	[32]	97.83	98.84
5	**Proposed System**	99.21	99.49

## Data Availability

Data are contained within the article.
